# The Comparative Effectiveness of Mepolizumab and Benralizumab in the Treatment of Eosinophilic Asthma

**DOI:** 10.3390/arm93040021

**Published:** 2025-06-20

**Authors:** Aleksandra Niemiec-Górska, Olga Branicka, Paula Olszewska, Sylwia Mielcarska, Joanna Glück, Barbara Rymarczyk, Radosław Gawlik

**Affiliations:** 1 Department of Internal Medicine, Allergology and Clinical Immunology, Faculty of Medical Sciences in Zabrze, Medical University of Silesia, 40-752 Katowice, Poland; 2 Doctoral School, Medical University of Silesia, 40-752 Katowice, Poland; 3 Department of Medical and Molecular Biology, Faculty of Medical Sciences in Zabrze, Medical University of Silesia, 40-752 Katowice, Poland

**Keywords:** eosinophilic asthma, biological treatment, mepolizumab, benralizumab

## Abstract

**Highlights:**

**What are the main findings?**

- A statistically significant decrease in eosinophil count, decrease in exacerbation rate, decrease in ACQ and increase in miniAQLQ were observed for both drugs. However, a statistically significant decrease in daily OCS dose and increase in FEV1/FVC were observed for mepolizumab, whereas no statistical significance was found for the above parameters for benralizumab. The latter two observations should be interpreted with caution due to the small study group.
- In general, there was no statistically significant difference in the level of improvement obtained between mepolizumab and benralizumab.

**What is the implication of the main finding?**

- Comorbidities, mainly CRwNS, and the drug regimen are the primary factors to consider when selecting a biologic drug.

**Abstract:**

Background: Severe asthma is associated with significant morbidity and risk of complications. Some patients, suffering from eosinophilic asthma, may benefit from biological therapies, especially anti IL-5 (anti-interleukin-5). The purpose of the study was to compare the efficacy evaluation of mepolizumab and benralizumab in the treatment of eosinophilic asthma. Methods: A retrospective, single-centre study including 59 patients with severe eosinophilic asthma treated with biologics (mepolizumab and benralizumab). Clinical outcomes, including peripheral blood morphotic characteristics, spirometry parameters, asthma control questionnaire (ACQ), mini-Asthma Quality of Life Questionnaire (mini-AQLQ) scores, daily oral corticosteroid use, body mass index, exacerbation rate, and exercise tolerance, were examined at the beginning and after 6 months of biological treatment. Results: A total of 38 patients were treated with mepolizumab and 21 with benralizumab. Significant improvements (*p* < 0.05) in eosinophil count, required daily dose of glucocorticoids, ACQ, mini-AQLQ scores, and exacerbation rate were observed in both groups after six months of treatment. There was no statistical difference (*p* < 0.05) in the abovementioned parameters between the groups. Conclusions: In patients with severe eosinophilic asthma, mepolizumab and benralizumab were associated with significant improvements in clinical state. Patients with type 2 asthma will benefit from the therapy with both anti-IL5 biologic drugs.

## 1. Introduction

Asthma is a persistent inflammatory disease affecting the respiratory tract, characterized by hyperactivity and the reversible obstruction of the airways. The main symptoms are wheezing, difficulties with breathing, coughing, and tightness in the chest [1]. Currently, there are two main classifications of asthma endotypes: T2-high and T2-low. Type T2-high, or eosinophilic asthma, is distinguished by an elevated number of eosinophils in both the bloodstream and sputum [2,3]. Type 2 inflammation occurs in approximately 70% of severe asthma patients and is promoted by a broad network of hyper-expressed cytokines, namely IL-4, IL-5, and IL-13, and several immune cells, including mastocytes, type-2 helper lymphocytes, type-2 innate lymphoid cells, basophils, and eosinophils [4,5].

Activated eosinophils exacerbate airway inflammation and induce bronchial mucosa damage by releasing various chemokines, cytokines, growth factors, and lipid mediators [6]. Growing scientific evidence suggests that eosinophils are crucial effector cells in airway remodelling [7]. Alterations in airway architecture observed in asthma include augmentation of smooth muscle mass, hyperplasia of goblet cells, and neovascularisation. The final outcome is an increased thickness of the bronchial wall, resulting in a diminished airway dimension, noticeable airway constriction, and a gradual deterioration of respiratory function [8,9].

Biological therapies offer a reprieve by targeting specific parts of the type 2 inflammatory pathway. Monoclonal antibodies targeting type 2 inflammatory pathways are available for the treatment of severe asthma, and have demonstrated efficacy to lower doses of oral glucocorticosteroid, reducing the frequency of exacerbations, and improving asthma control and lung function [4,10]. Characterizing the asthma endotype and phenotype of patients with severe asthma and determining which treatment would be more appropriate for a particular patient is an essential step towards personalized treatment. The main classes of monoclonal antibodies used in severe asthma treatment are anti-IgE (anti-immunoglobulin E) (omalizumab), anti-IL5/anti-IL5R (anti-interleukin 5 or anti-the receptor of interleukin-5) (mepolizumab, reslizumab, and benralizumab), anti-IL4R (anti-the receptor for interleukin 4) (dupilumab), and anti-TSLP (anti-thymic stromal lymphopoietin) (tezepelumab) [11].

Both mepolizumab and benralizumab target IL-5, an interleukin that plays a crucial role in the activation, maturation, and survival of eosinophils [2]. However, the two drugs have different mechanisms of action. Mepolizumab binds to soluble IL-5, preventing it from binding to the IL-5 receptor on eosinophils, thereby suppressing eosinophil proliferation and activation [12]. In turn, benralizumab is a monoclonal antibody that has a distinctive dual activity. The drug specifically binds to IL-5Rα via the Fab fragments, which effectively inhibits the interaction between IL-5 and its receptor. Moreover, benralizumab interacts with the FcγIIIRa receptor on NK cells via the Fc constant region, resulting in the promotion of ADCC (antibody-dependent cellular cytotoxicity) induced apoptosis in eosinophils [13].

Mepolizumab and benralizumab are biologic drugs approved for the management of severe eosinophilic asthma. Despite their widespread use, direct comparative studies between the two pharmaceuticals are incomplete, forcing physicians to depend on indirect comparisons for therapeutic decision-making. This issue is clinically relevant, as the selection of treatment can influence various factors, including efficacy, compliance of patients, and cost-effectiveness [14,15]. As a result, understanding the relative efficacy and safety of mepolizumab and benralizumab is essential for evidence-based asthma treatment.

This study aims to evaluate benralizumab and mepolizumab in the treatment of severe eosinophilic asthma, considering spirometry, blood morphology parameters, quality of life and asthma control questionnaires, as well as improvements in exercise tolerance.

## 2. Materials and Methods

### 2.1. Study Design and Participants

This retrospective, single-centre study was performed at the Department of Internal Diseases, Allergology and Clinical Immunology in the University Clinical Hospital named K. Gibińskiego Medical University of Silesia in Katowice, Poland. The study enrolled 59 patients: 38 of them started treatment with mepolizumab between July 2018 (in-clinic introduction) and December 2023 and 21 of them with benralizumab between December 2019 (in-clinic introduction) and December 2023. Reslizumab was omitted from our study due to its unavailability in Poland. In turn, dupilumab was excluded from our analysis because of the small number of patients treated with this drug at our clinic. The study included patients who met the criteria for initiating the biologic treatment of severe asthma with benralizumab or mepolizumab in accordance with the requirements that are mandatory in Poland. The eligibility criteria for treatment with benralizumab and mepolizumab according to the guidelines of the Polish Ministry of Health are as follows:

- Blood eosinophil count ≥ 350/µL in the last 12 months or ≥150 cells/μL; if systemic, glucocorticosteroids at a dose ≥ 5 mg per day had to be taken systematically in the 6 months prior to the qualification and the cumulative annual dose of oral glucocorticosteroids was ≥1.0 g (calculated as prednisone) due to a lack of asthma control.
- The need for high doses of inhaled glucocorticosteroids (>1000 mcg of beclomethasone dipropionate per day or another inhaled glucocorticosteroid at an equivalent dose determined according to the current guidelines from The Global Initiative for Asthma (GINA)) in combination with another asthma control medication.
- Two or more exacerbations in the past year that required systemic glucocorticosteroids or an increase in their dose for more than three days in people who use them chronically.
- Meeting at least 2 of the following criteria:
  (a) Symptoms of uncontrolled asthma (lack of asthma control in the ACQ asthma control questionnaire > 1.5 points);
  (b) Hospitalization in the last 12 months due to asthma exacerbation;
  (c) A life-threatening asthma attack incident in the past;
  (d) Persistent airway obstruction (first-second expiratory volume FEV1 < 80% of normal value or diurnal variation in peak expiratory flow PEF > 30%);
  (e) Impaired quality of life due to asthma (mean score on the asthma quality of life test mini-AQLQ < 5.0 points).
- Exclusion of other hypereosinophilic syndromes.
- Non-smoking.
- Exclusion of other clinically relevant pulmonary diseases [16].

Despite the fact that the general eligibility criteria for both drugs are the same, in our clinic, patients suffering from severe asthma and chronic rhinosinusitis with nasal polyps (CRwNP) were more frequently qualified for treatment with mepolizumab compared to benralizumab. In turn, benralizumab was preferentially selected for patients with severe asthma who faced challenges attending frequent hospitalisations due to logistical factors, such as distant locations from the clinic, extensive work obligations or mobility impairments.

According to the summary of product characteristics, mepolizumab was administered at a dose of 100 mg every four weeks, while benralizumab was initially administered three times every four weeks at a dose of 30 mg, and subsequently every eight weeks [17,18]. Patients were evaluated twice: first at point 0 (before therapy started) and then at point 1 (six months into treatment).

### 2.2. Exclusion Criteria

The criterion for exclusion from the study was discontinuation of treatment before the follow-up point after 6 months.

### 2.3. Assessment of Clinical Efficacy

The assessment of asthma control was performed by the asthma control questionnaire (ACQ-7), which consists of seven items. These items include five questions related to symptoms, one question concerning the use of rescue bronchodilators, and one question about FEV1. Each item is scored on a seven-point scale, ranging from 0 (indicating complete control) to 6 (indicating maximum impairment) [19]. The ACQ score is calculated as the average of the questions included. A change in ACQ of 0.5 is considered to be the minimum significant difference.

Quality of life was assessed using the miniAQLQ, an asthma-related quality of life questionnaire. The mini-AQLQ is a 15-item list that is scored on a 7-point scale, with 1 representing severe impairment and 7 representing no impairment [20]. The quality of life is positively correlated with a higher score on the questionnaire. The minimal significant difference for a change in mini-AQLQ is 0.5.

Based on the patient’s medical history, the amount of inhaled medication (ICS (inhaled corticosteroids), LABA (long-acting inhaled β2-mimetics), LAMA (long-acting drugs of the muscarinic receptor antagonist group), SABA (short-acting inhaled β2-mimetics), oral glucocorticosteroid dosage, and other medications used to manage asthma symptoms (LTRA) (antileukotrienes drugs) were also evaluated.

Additionally, after 6 months of treatment, patients subjectively assessed improvements in exercise tolerance using a four-point Likert scale. The evaluation was retrospective and single-stage. At the first follow-up point (point 1) patients answered the question —how would you rate the improvement in exercise tolerance during biological therapy? It was possible to provide one of the following answers: ‘−1’—worsening of exercise tolerance; ‘0’—no change; ‘1’—slight improvement; and ‘2’—noticeable improvement. Responses with scores of 1 or 2 were considered to be an improvement. The number assigned to the particular response provided by the patient was recorded in the database.

### 2.4. Laboratory Tests

All the patients enrolled in the study underwent routine complete blood tests at the time of admission for biological therapy (point 0) and repeated after 6 months (point 1). Samples were collected using a test tube containing an anticoagulant (EDTA-ethylenediaminetetraacetic acid). The Sysmex XN-350 haematology analyser, manufactured by Sysmex Europe Corporation (Norderstedt, Germany), was used to measure and record the absolute counts of neutrophils, lymphocytes, eosinophils, platelets, and monocytes.

In addition, CRP was determined at point 0 and point 1. Whole blood samples were allowed to coagulate at ambient temperature for 30 min. The supernatant was centrifuged at 5000× *g* for 10 min. Subsequently, CRP was determined in serum samples using a Cobas PRO analyser (Roche Diagnostics GmbH, Mannheim, Germany). The cut-off point for the normal value was established as 5 mg/L. ESR, in turn, was determined using an analyser manufactured by DIESSE Diagnostica Senese S.p.A (Monteriggioni, Italy).

Allergy was diagnosed on the basis of allergen-specific IgE antibody determinations in the serum of patients using the Polycheck method. A concentration > 0.35 kU/L was considered a positive result. A second method of allergy diagnosis was skin prick tests. A positive test result indicating IgE-mediated hypersensitivity is considered to be an urticarial wheal of at least 3 mm in diameter, and should be surrounded by erythema.

### 2.5. Spirometry

Spirometry was routinely performed in patients before the administration of the biologic treatment, prior to the use of bronchodilator (point 0), and after 6 months of treatment (point 1). The test was performed by the nurses in the Allergology Department using a Ganshorn Medizin Electronic spirometer (SpiroScout). The following parameters were evaluated FEV1 (forced expiratory volume), FEV1%, FVC (forced vital capacity), and FEV1/FVC.

### 2.6. Evaluation

The patients were assessed before the start of therapy (point 0) and after 6 months of treatment (point 1).

#### 2.6.1. Laboratory Parameters

The parameters included were leukocyte, eosinophil, neutrophil, monocyte, and platelet counts, as well as inflammatory markers—CRP and ESR. Determinants were assessed at point 0 (before the initiation of biological treatment) and point 1 (after 6 months of therapy).

The eosinophil count in the study may have been lower than 350/microlitre because the morphological result on the day of admission was taken into account. A result of more than 350 eosinophils in the last 12 months (historical determination) is acceptable to initiate biological treatment as well. We assumed that all the determinations in the study were performed on the same laboratory equipment.

#### 2.6.2. Spirometry Results

Further parameters assessed were pulmonary function test results (FEV1, FEV1%, FVC, and FEV1/FVC). Spirometry was performed at point 0 and at point 1.

#### 2.6.3. Questionnaires

The patients completed the validated ACQ and miniAQLQ questionnaires in Polish at point 0 and at point 1. The methods of questionnaire evaluation are described in Section 2.2.

#### 2.6.4. Oral Glucocorticosteroid Dose

The daily doses of oral glucocorticosteroid used by the patients at baseline 0 and checkpoint 1 were assessed. The study considered the current OCS dose used on the day of assessment, not doses generally applied during asthma exacerbations.

#### 2.6.5. Body Mass Index

Body mass index (BMI), calculated as body weight divided by height to the power of two, was also assessed. BMI was assessed twice, analogous to the parameters listed above. The normal range of BMI was 18.5–24.9. Overweight was found in patients with BMI 25.0–29.9, while obesity was diagnosed in patients with BMI > 30.

#### 2.6.6. Exacerbation Rate

The cumulative number of exacerbations in the 12 months preceding eligibility for biological therapy was evaluated according to the regulations of the Polish drug programme for the treatment of severe asthma. It is defined as the exacerbation rate at point 0. The frequency of exacerbations from the initiation of biological therapy to the checkpoint (point 1) was subsequently evaluated.

### 2.7. Ethics

The study protocol was reviewed and approved by the Bioethics Committee at the Medical University of Silesia in Katowice (BNW/NWN/0052/KB/217/24; 26 September 2024), and was in accordance with the ethical principles for human experimentation initiated by the Declaration of Helsinki. The study was exempt from the requirement to obtain written informed consent from the patients due to the retrospective nature of the study and the need for informed consent was waived. However, all the patients signed an informed consent before starting biological treatment. Patient confidentiality was maintained in the analysis, and the analysis was performed using an anonymized dataset.

### 2.8. Statistical Analysis

The results are expressed as absolute numbers and percentages for frequencies, and mean ± standard deviations (if normally distributed) or median and interquartile range (if not normally distributed). Normality was checked using the Shapiro–Wilk test. The Wilcoxon matched-pairs signed-rank test was used to compare outcomes between both time-points, and the Mann–Whitney *U* test was used to compare both groups. All the analyses were performed with a software package (The STATISTICA 13.3, StatSoft Krakow, Poland). P-values less than 0.05 were considered significant.

## 3. Results

### 3.1. Clinical and Demographic Characteristics

**Figure 1:**
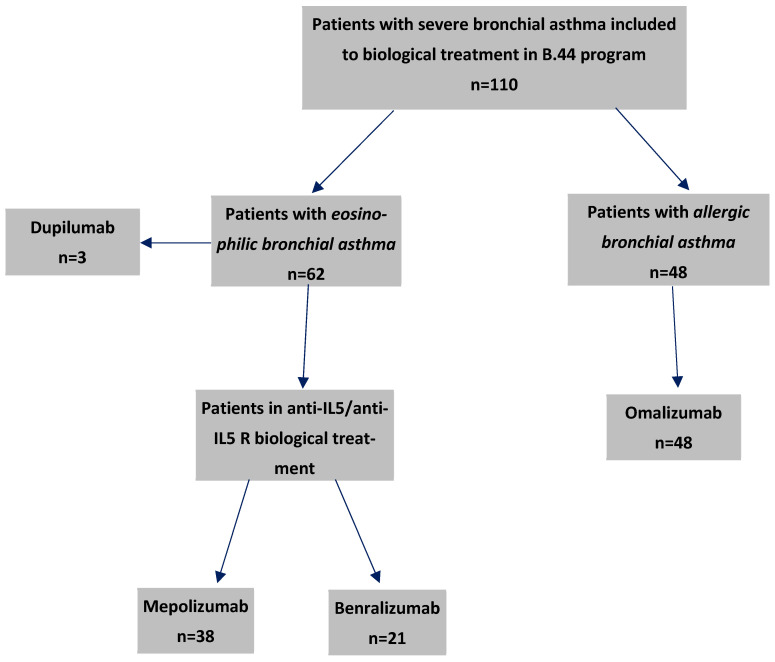
Flow chart of patients on biological treatment for severe asthma with particular drugs at our clinic who started therapy before the end of 2023.

Between the years 2018 and 2023, 59 patients with severe bronchial asthma (46 female, 78%) and a mean age of 54.1 years (range 23–80) met the inclusion criteria and underwent the biological treatment (Figure 1). Of these, 26 patients (44%) had allergic rhinitis (seasonal or perennial). The groups of patients treated with mepolizumab and benralizumab were comparable in terms of sex, age, smoking habits, inhalant allergies, and comorbidities as shown in Table 1.

### 3.2. Mepolizumab

The study comprised a cohort of 38 individuals who underwent mepolizumab therapy. Of these, 32 were women (84.2%). The average age of the patients analysed was 52.8 years. The majority of the patients exhibited a condition of being overweight (44.7%) or obese (21.1%). Additionally, 15 patients (39.5%) had chronic rhinosinusitis with nasal polyps, 5 (13.2%) osteoporosis, 3 (7.9%) post-steroid diabetes, and 14 (36.8%) hypertension. Half of the patients had a history of allergy, suffering from seasonal or perennial rhinitis. Furthermore, seven (18.4%) of the patients were former tobacco smokers (on average 4.7 pack-years) and none were current smokers.

All the patients were treated with high doses of ICS and LABA; additionally, LAMA was used by 4 patients and LTRA by 17 patients. SABA as a reliever was used by 23 (60.5%) patients, and the others used supplementary doses of ICS + LABA (in addition to the prescribed basal dose) or SAMA. After 6 months of biological treatment, ICS + LABA dose was reduced twice in one patient and four times in another one. In the rest of the patients, the dose of ICS+LABA in chronic treatment did not change.

**Figure 2:**
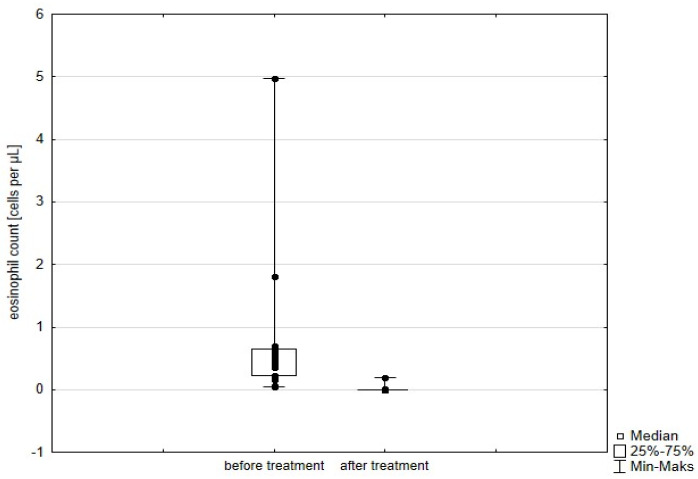
Eosinophil count in patients treated with mepolizumab. Wilcoxon test for paired samples, *p* < 0.01.

**Figure 3:**
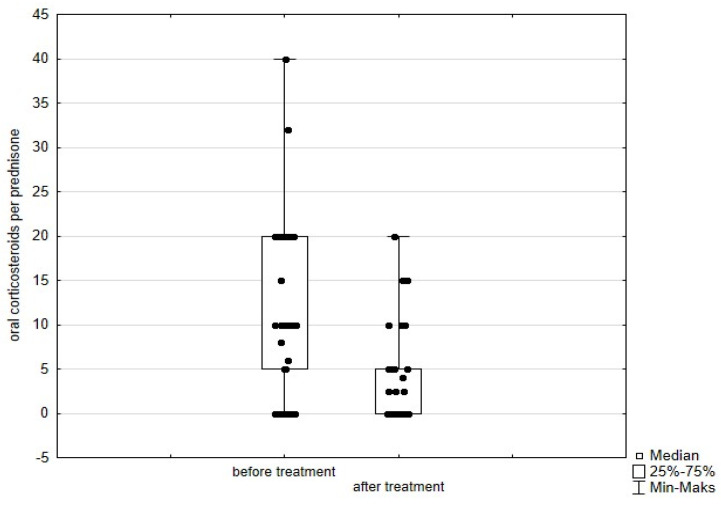
Daily dose of oral corticosteroids per prednisone in patients treated with mepolizumab. Wilcoxon test for paired samples, *p* < 0.01.

**Figure 4:**
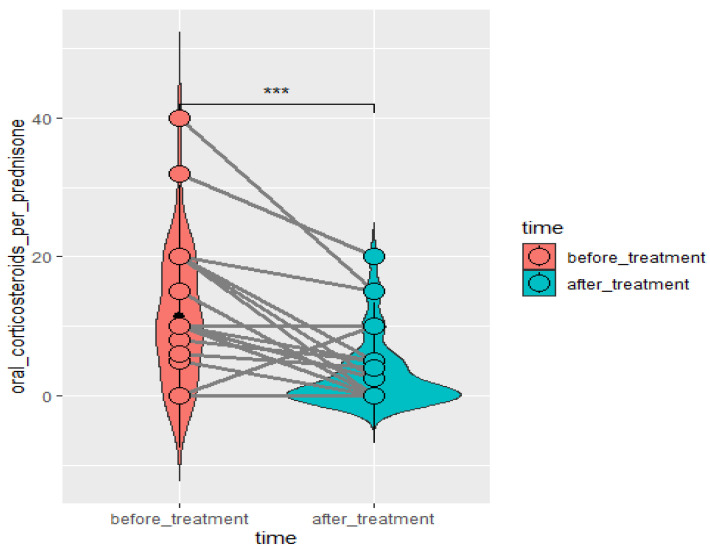
Change in daily OCS dose in time in patients treated with mepolizumab. Wilcoxon test for paired samples, *p* < 0.01. ***—*p* < 0.01.

**Figure 5:**
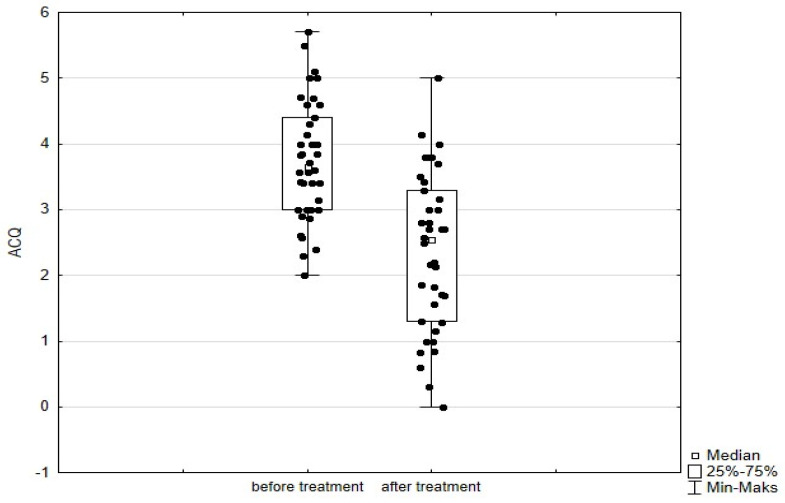
ACQ scores in patients treated with mepolizumab. Wilcoxon test for paired samples, *p* < 0.01.

**Figure 6:**
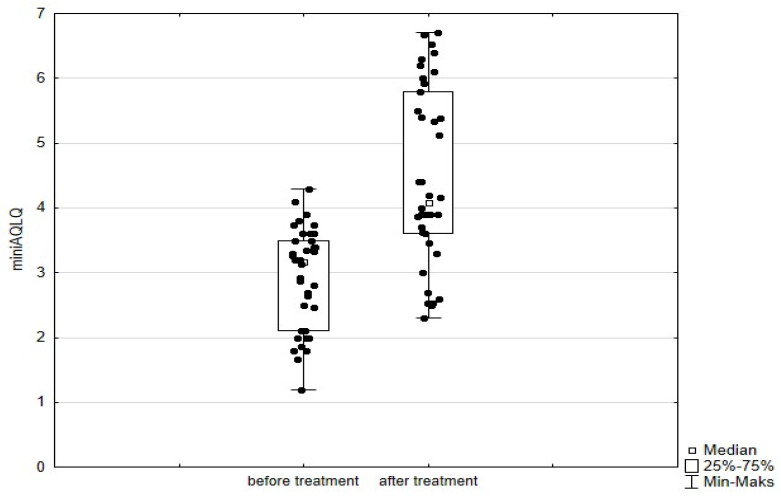
miniAQLQ questionnaire scores in patients treated with mepolizumab. Wilcoxon test for paired samples, *p* < 0.01.

**Figure 7:**
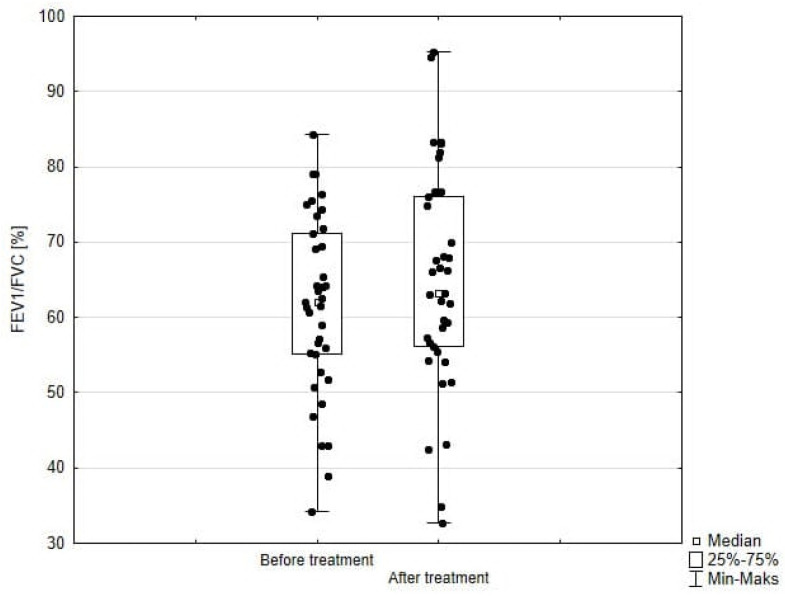
FEV1/FVC in patients treated with mepolizumab. Wilcoxon test for paired samples, *p* = 0.03.

**Figure 8:**
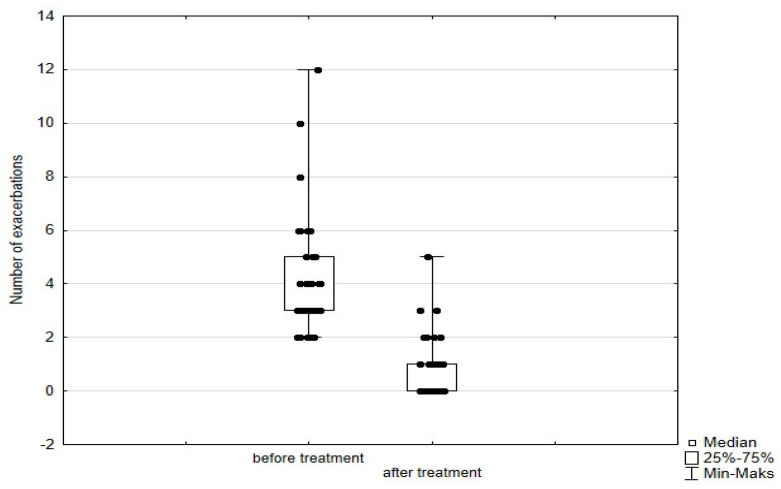
Number of exacerbations in patients treated with mepolizumab. Wilcoxon test for paired samples, *p* < 0.01.

Significant improvement (*p* < 0.05) was found in several parameters throughout the 6-month observation, including a drop of eosinophil (Figure 2), a reduction in oral glucocorticosteroid dose—from a median dose of 10 mg per prednisone to complete withdrawal. (Figure 3 and Figure 4), a decrease in ACQ (Figure 5) and an increase in miniAQLQ (Figure 6) questionnaire scores, and an increase in spirometry parameters indicative of obstruction (FEV1/FVC) (Figure 7). A decrease in the number of exacerbations was also observed (Figure 8). In contrast, there was no statistically significant difference in the number of leukocytes, lymphocytes, platelets, neutrophils, inflammatory measures (CRP-c-reactive protein, ESR-red blood cell sedimentation reaction), BMI, and absolute values of FEV1 and FVC.

Furthermore, the patients rated the improvement in exercise tolerance during treatment using a Likert scale. A total of 23 patients (60.5%) noted an improvement in exercise tolerance, of which 7 patients (18.4%) rated the improvement as noticeable. None reported a reduction in exercise capacity.

### 3.3. Benralizumab

The study comprised a cohort of 21 individuals treated with benralizumab. Of these, 7 individuals were men (33.3%) and 14 women (66.7%). The average age of the patients analysed was 55.8 years. Obesity was found in nine patients (42.9%), while six (28.6%) were overweight. Furthermore, 5 patients (23.1%) had chronic rhinosinusitis with nasal polyps, 1 (4.8%) osteoporosis, 4 (19.0%) post-steroid diabetes, and 12 (57.1%) arterial hypertension. Out of the total number of patients, seven individuals (33.3%) suffered from an allergic disease: seasonal or perennial rhinitis. Additionally, four patients (19.0%) were former smokers (on average 5.1 pack-years), and currently none of them smoke.

All the patients were administered high dosages of ICS and LABA; 2 patients used LAMA, and 13 received LTRA. SABA was used as a reliever by 12 patients (57.1%), while the rest received additional doses of ICS + LABA or SAMA. After six months of biological therapy, the ICS + LABA dosages in chronic treatment for all the patients remained unchanged.

**Figure 9:**
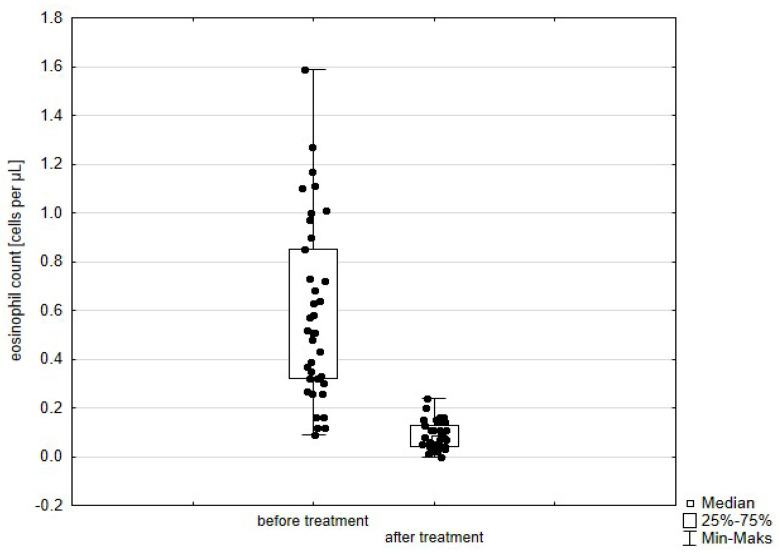
Eosinophil count in patients treated with benralizumab. Wilcoxon test for paired samples, *p* < 0.01.

**Figure 10:**
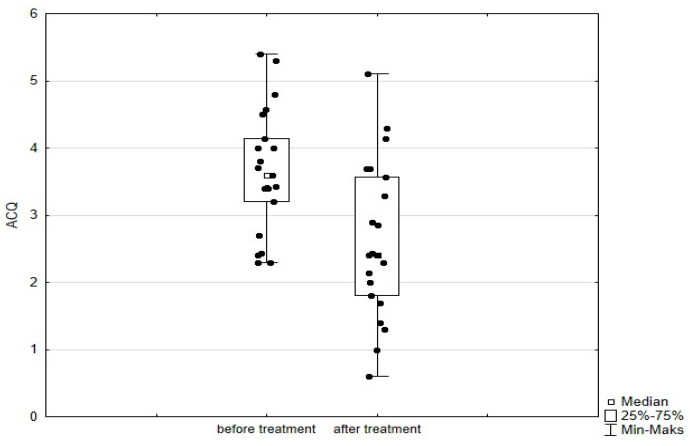
ACQ scores in patients treated with benralizumab. Wilcoxon test for paired samples, *p* < 0.01.

**Figure 11:**
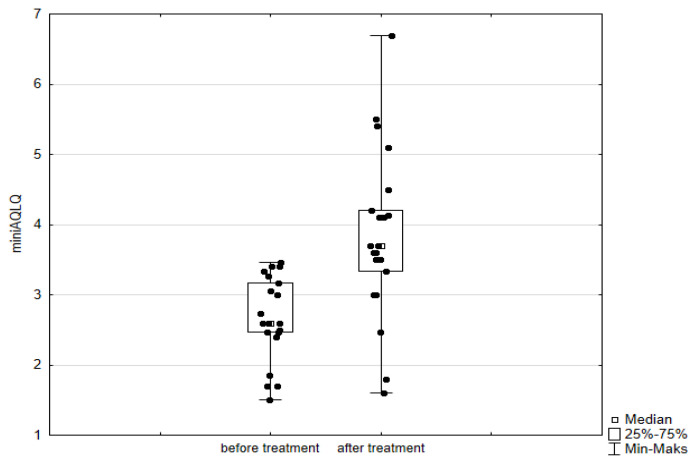
miniAQLQ questionnaire scores in patients treated with benralizumab. Wilcoxon test for paired samples, *p* < 0.01.

**Figure 12:**
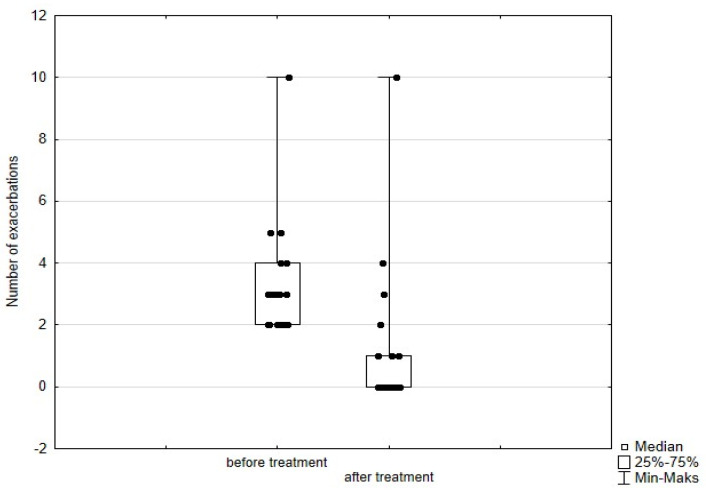
Number of exacerbations in patients treated with benralizumab. Wilcoxon test for paired samples, *p* < 0.01.

**Figure 13:**
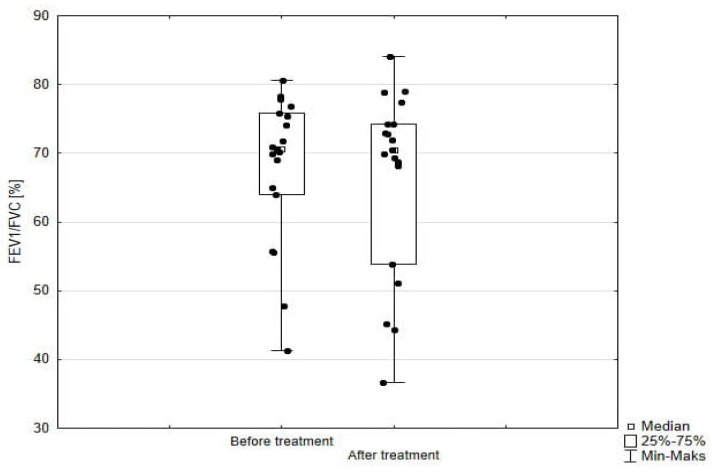
FEV1/FVC in patients treated with benralizumab. Wilcoxon test for paired samples, *p* = 0.69.

**Figure 14:**
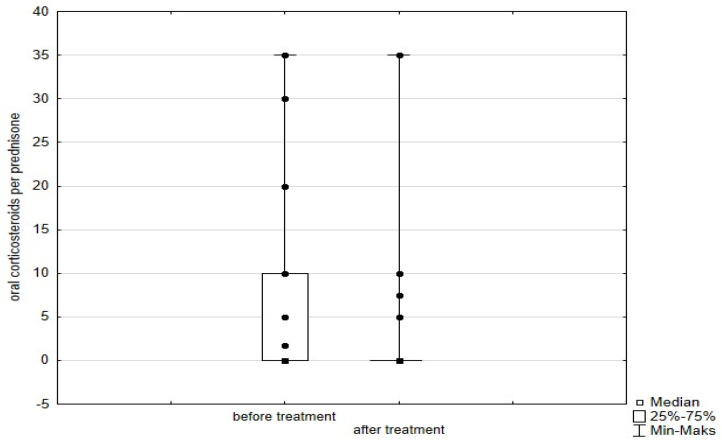
Daily dose of oral corticosteroids per prednisone in patients treated with benralizumab. Wilcoxon test for paired samples, *p* = 0.0506.

**Figure 15:**
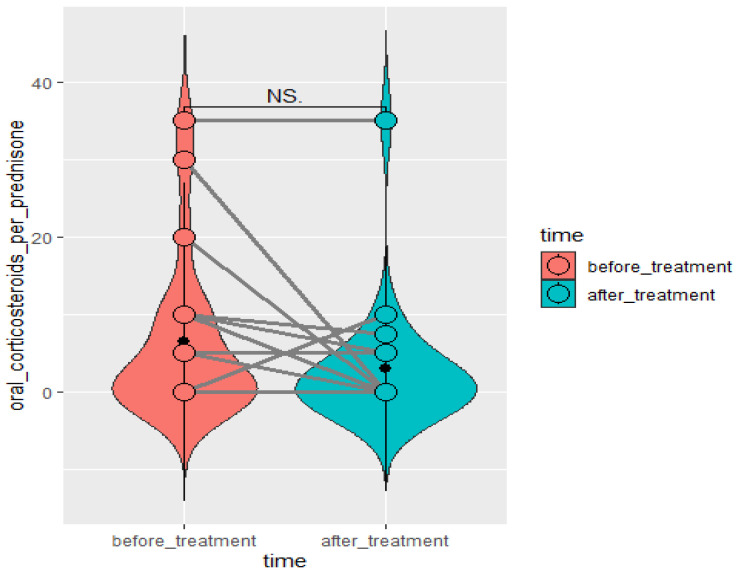
Change in daily OCS dose in time in patients treated with benralizumab. Wilcoxon test for paired samples, *p* = 0.0506. NS—not statistically significant.

Statistically significant improvements (*p* < 0.05) were seen across numerous parameters after 6 months of benralizumab therapy. These parameters include a decline in leukocytes, and neutrophils, and a substantial reduction in eosinophils (Figure 9), lymphocytes, and platelets. Furthermore, a decrease in ACQ (Figure 10) and increase in miniAQLQ (Figure 11) questionnaire scores, and a decrease in exacerbation rate (Figure 12) were observed. However, there were no notable variations seen in the monocyte count, inflammatory markers (CRP and ESR), BMI, and spirometry measurements (FEV1, FEV1%, FVC, and FEV1/FVC) (Figure 13). There was also no statistical significance (*p* = 0.0506) for OCS dose reduction. (Figure 14 and Figure 15).

Moreover, analogous to mepolizumab, the patients assessed improvements in exercise tolerance on a Likert scale. A total of 12 patients (57.1%) achieved an improvement in exercise tolerance during therapy, of which a noticeable improvement was declared by 4 patients (19.0%). None reported a reduction in exercise capacity as well.

### 3.4. Comparison Between Mepolizumab and Benralizumab

Table 2 presents the values for each parameter related to mepolizumab and benralizumab at point 0 (before biological treatment) and point 1 (at follow-up point), together with the statistical significance of these values at the specified points.

Both mepolizumab and benralizumab demonstrated substantial therapeutic benefits, including a reduction in eosinophil count and ACQ scores. In addition, reduced use of rescue drugs (SABA or additional doses of ICS + LABA) was also observed in both subgroups. A noticeable improvement in patients’ quality of life was observed, as evidenced by the elevated scores on the miniAQLQ questionnaire, along with patients’ reports of improved exercise tolerance. A statistically significant decrease in daily OCS dose and an increase in FEV1/FVC was observed for mepolizumab, whereas no statistical significance was found for the abovementioned parameters for benralizumab.

Both biologics led to a significant reduction in eosinophil count (*p* < 0.05). However, there was no statistical difference in the average decrease between individuals receiving mepolizumab and those treated with benralizumab. However, a significantly higher percentage of patients (90.5%) who received benralizumab had undetectable levels of eosinophils after treatment, whereas no patients treated with mepolizumab had the same result. Overall, there was no statistically significant difference between the two drugs in the level of improvement achieved.

In addition, it is important to note that we did not observe any side effects in our patients during the six-month therapy with both drugs, which indicates the high safety of these biologicals.

## 4. Discussion

The aim of our study was to find out whether there is a difference in effective assessment: one drug produced better clinical outcomes than the other in the biological treatment of eosinophilic asthma. It was a preliminary analysis of the markers and clinical and laboratory parameters of patients with severe asthma after 6 months of biological therapy.

The analysis of clinical efficacy is interesting because the criteria for starting treatment with both drugs are the same, at least in Poland. The choice of therapy is ultimately made by the physician, based on an analysis of multiple factors, i.a., comorbidities or patient preferences. Comparing the two biologics in real-world conditions is crucial since it provides insight into their performance outside of controlled clinical trials. Real-life studies account for variability in patients’ groups, concerning comorbidities, age, smoking habits, etc., offering a more precise picture of treatment outcomes. This enables physicians to make informed decisions based on individual patient’s needs, improving the overall quality of medical care [21,22].

One of the criteria for drug selection was the coexistence of severe asthma with chronic rhinosinusitis with nasal polyposis (CRwNP). The biologic drugs currently approved by the FDA (Food and Drug Administration) for the treatment of CRwNP are dupilumab, mepolizumab and omalizumab. Benralizumab does not have approval for this indication [23]. Cai et al. [24] found that benralizumab had the lowest efficacy in reducing nasal symptoms and SNOT-22 (Sino-Nasal Outcome Test) questionnaire scores relative to other biologics, including mepolizumab. However, research has indicated that benralizumab may be beneficial in the treatment of CRwNP [25,26]. Han et al. [27] highlighted that mepolizumab has been shown to reduce nasal polyp size and improve sinus-related symptoms in patients with asthma and CRwNP, supporting its role in patients with both conditions. In our department, the patients diagnosed with severe asthma and CRwNP were more likely to be qualified for therapy with mepolizumab rather than benralizumab.

In addition, the study found that both biologic medicines were highly effective in clinical practice. Both drugs show high efficacy in steroid-dependent asthma, allowing a reduction in oral glucocorticosteroid doses. OCS has multiple side effects, including post-steroidal diabetes or osteoporosis [28]. Preventing post-steroid complications improves general health conditions and lowers patients’ treatment costs [29].

Studies have shown that both biologic IL-5 therapy effectively reduce the need for oral glucocorticosteroids, improve asthma control, and diminish the presence of eosinophils in the blood [30,31]. Most important from the patient’s perspective was the improvement of physical abilities and the removal of previous limitations in this area. Despite the existence of several indirect systematic reviews and meta-analyses comparing the effects of different biologics, the evidence remains inconclusive. Menzella et al. [32] demonstrated that benralizumab has potential superiority over mepolizumab in decreasing the use of oral glucocorticosteroids. In our study, we observed the opposite results. Nevertheless, they should be interpreted with caution due to the small study group. Additionally, Busse et al. [33] reported that mepolizumab had a stronger correlation with better asthma control in comparison to benralizumab. According to Kayser et al. [34], mepolizumab and benralizumab resulted in significant improvements in lung function, as evidenced by an increase in median first-second expiratory volume (FEV1). In addition, treatment provided a notable improvement in ACT scores and a reduction in mean daily OCS dose. Exacerbation rates were considerably reduced, regardless of the drug administered. In general, alterations were comparable after 6 and 12 months of treatment [34]. Cabon et al. and Bourdin et al. [35,36] published similar findings in their meta-analyses of clinical trials, demonstrating that reslizumab, mepolizumab, and benralizumab had comparable effectiveness in enhancing lung function and reducing exacerbations. Our study showed a statistically significant improvement in FEV1/FVC during mepolizumab treatment, but no such pattern was found with benralizumab therapy. However, Vitale et al. [37] observed an improvement in FEV1/FVC during benralizumab therapy after 6 months of treatment, as well as at subsequent follow-up points. In fact, our analysis did not show statistically significant differences between mepolizumab and benralizumab for any variables mentioned above.

Our investigation aimed to evaluate the alterations in blood morphology and inflammatory markers. Determinations are cost-effective to conduct and are required for the qualification and monitoring of biological treatment, at least under Polish conditions. Furthermore, relying upon prior publications concerning the significance of peripheral blood morphology determinants in predicting treatment responses to biologic drugs and in distinguishing between asthma with NERD and asthma without NERD, we performed an evaluation of the parameters mentioned above [38,39]. Our investigation revealed no statistically significant differences, with the exception of a decrease in eosinophil counts in blood morphology. Ghassemian et al. [2] found that while there was no significant difference in the degree of reduction in peripheral eosinophil counts between benralizumab and mepolizumab, benralizumab was able to reduce eosinophil levels to zero in almost all the patients. Our results confirm this observation. In contrast, the results of inflammatory parameters—CRP and ESR—were within the normal range both before and after 6 months of treatment. This indirectly indicates the safety of these drugs and that, in short-term follow-up, these drugs do not increase the risk of infection or inflammatory status.

Langton et al. [40] observed in a large group of severe eosinophilic asthmatics that both mepolizumab and benralizumab improved asthma control. However, benralizumab treatment appeared to be significantly more effective than mepolizumab in reducing exacerbations and improving FEV1.

A study performed by Franceschi et al. [41] evaluated improvements in exercise tolerance in patients receiving benralizumab and mepolizumab. The researchers used plethysmography, blood gas analysis, and cardiopulmonary exercise test analysis. Maximum load, peak oxygen consumption, respiratory capacity, respiratory rate, and tidal volume did not significantly alter over 3 months [41]. Unfortunately, we were unable to perform plethysmography or blood gas analysis, so we evaluated the patients’ enhancement in exercise tolerance via a questionnaire. Nevertheless, we are aware that analyses based on measurable (objective) parameters are of greater scientific value than those depending on the subjective reports of the patient.

Considering the comparable effectiveness of mepolizumab and benralizumab treatments in pharmacological and clinical aspects, non-medical factors may influence the decision-making process. The overall costs of biologic therapy comprise the cost of purchasing the biologic and the expense of its administration, determined by the expected number of annual administrations and the cost associated with each administration visit. According to the analysis for US conditions, benralizumab had the lowest overall biologic cost: USD 72,064 for 2-year and USD 138,980 for 4-year intervals. The marginal cost difference between benralizumab and mepolizumab for biologic expenses revealed that mepolizumab was USD 8957 more expensive over a 2-year period and USD 23,061 more pricey over a 4-year period [15]. Another aspect of selecting a biologic drug is the administration schedule. Benralizumab is delivered monthly for the initial three months, followed by bi-monthly administration, resulting in a total of eight visits annually. Mepolizumab is administered monthly, resulting in a total of 12–13 visits per year [17]. This is crucial for elderly patients, individuals with physical disabilities, and those employed or living at a considerable distance from a reference centre, particularly when the medication is not provided for home administration [42].

The research revealed no significant differences between the biologics in terms of the parameters evaluated in the study in short-term observation. It is important to consider the limitations of this investigation, which include the small sample size, the uneven distribution of patients across the groups, the single-centre nature of the study, and the lack of a control group.

An essential constraint of this study, as with most real-life studies involving biological therapy, is the absence of a placebo control group. The absence of a control group reduces the reliability of the comparison of observed results. It also has limitations associated with retrospective data collection, such as the lack of significant values for some patients due to deficiencies in medical records, and the inability to increase or equalize the sample size [2,43,44].

The results of the trials comparing the drugs may not be representative because of the relatively small numbers of patients (38 for mepolizumab and 21 for benralizumab). Furthermore, the cohorts were unequal. The majority of patients in our study were female. Nonetheless, scientific research indicates that women are more prone to asthma and have an elevated risk of severe disease compared to males [45]. Additionally, the follow-up time during the study was relatively short. Only one comprehensive assessment of patients was performed within 6 months. As a result, there was no apparent trend of improvement. On the other hand, certain variables, such as a recent infection, could have impacted spirometry, laboratory test results, or questionnaire scores.

We will follow the observation of treated patients for the next months which will provide us with further information on the effectiveness of treating severe asthma with both biologics. This could be helpful in precise treatment of severe asthma.

## 5. Conclusions

In conclusion, both mepolizumab and benralizumab exhibit comparable clinical efficacy in individuals suffering from severe eosinophilic asthma in relation to blood eosinophil number, daily dose of glucocorticoids, asthma control, quality of life, exacerbation rate and exercise tolerance after six months of treatment. However, drug therapies differ in the frequency they are administered.

## Figures and Tables

**Table 1:** Demographic and clinical features of patients at the beginning of the study (point 0).

	Total	Mepolizumab	Benralizumab	*p*-Value
Patients, n (%)	59	38 (64.4)	21 (35.6)	0.36
Gender (Female), n (%)	46 (78) (78)	32 (84.2)	14 (66.7)	0.12
Age, years (mean ± SD)	54.1 ± 13.1	52.8 ± 11.4	55.8 ± 15.4	0.83
Obesity, n (%)	17 (28.8)	8 (21.1)	9 (42.9)	0.08
Overweight, n (%)	25 (42.4)	17 (44.7)	6 (28.6)	0.11
Ex-smokers, n (%)	11 (18.6)	7 (18.4)	4 (19.0)	0.55
Inhalant allergies, n (%)	28 (47.4)	19 (50.0)	7 (33.3)	0.38
Nasal polyps, n (%)	20 (33.9)	15 (39.5)	5 (23.1)	0.22
GERD, n (%)	7 (11.9)	5 (13.2)	2 (9.5)	0.68
Diabetes, n (%)	7 (11.9)	3 (7.9)	4 (19.0)	0.20
Osteoporosis, n (%)	6 (10.2)	5 (13.2)	1 (4.8)	0.31
Arterial Hypertension, n (%)	36 (61.0)	14 (36.8)	12 (57.1)	0.32
ICS, n (%)	59 (100)	38 (100)	21 (100)	1.00
LABA, n (%)	59 (100)	38 (100)	21 (100)	1.00
SABA, n (%)	35 (59.3)	23 (60.5)	12 (57.1)	0.65
LAMA, n (%)	6 (10.2)	4 (10.5)	2 (9.5)	0.90
LTRA, n (%)	30 (50.1)	17 (44.7)	13 (61.9)	0.21
OCS maintenance > 6 months, n (%)	18 (30.5)	13 (34.2)	5 (23.8)	0.41

n—number of patients; GERD—gastrointestinal reflux disease; ICS—inhaled corticosteroids; SABA—short acting β_2_ agonist; LABA—short acting β_2_ agonist; LAMA—long-acting muscarinic antagonist; LTRA—leukotriene receptor antagonist; OCS—oral corticosteroid.

**Table 2:** Median values and interquartile ranges of complete blood, spirometry parameters, asthma control tests, quality of life questionnaires, daily oral glucocorticosteroid use, and exacerbation rate in patients with severe bronchial asthma were assessed in point 0 and point 1; *p*-values show the difference between mepolizumab and benralizumab at each visit.

	Mepolizumab (Point 0)	Benralizumab (Point 0)	*p*-Value (Point 0)	Mepolizumab (Point 1)	Benralizumab (Point 1)	*p*-Value (Point 1)
Leukocytes, 10^3/^µL	7.80 (6.69–7.13)	9.00 (7.13–9.01)	0.17	6.64 (5.12–8.92)	7.75 (5.71–6.85)	0.58
Lymphocyte count, 10^3/^µL	2.12 (1.50–2.64)	2.08 (1.52–3.38)	0.71	2.04 (1.45–2.30)	1.72 (1.43–2.35)	0.56
Eosinophil count, 10^3/^µL	0.51 (0.32–0.85)	0.49 (0.22–0.65)	0.63	0.08 (0.04–0.13)	0.00 (0.00–0.00)	0.00
Monocytes count, 10^3/^µL	0.64 (0.41–0.87)	0.68 (0.51–0.79)	0.61	0.56 (0.42–0.76)	0.60 (0.49–0.68)	0.70
Neutrophil count, 10^3/^µL	4.39 (3.00–5.69)	5.49 (4.20–8.39)	0.08	4.25 (2.86–5.64)	4.40 (3.51–5.84)	0.43
Platelet count, 10^3/^µL	276 (226–316)	277 (244–336)	0.42	266 (249–344)	248 (225–278)	0.14
CRP [mg/L]	1.90 (0.90–6.90)	1.40 (0.90–5.30)	0.56	1.95 (0.70–6.00)	1.70 (0.90–4.80)	0.93
ESR [mm/h]	11.00 (4.00–17.00)	9.00 (5.00–17.00)	0.97	9.00 (5.00–20.00)	9.00 (5.00–12.00)	0.59
FVC [L]	2.47 (1.85–3.17)	2.67 (1.95–3.46)	0.60	2.47 (1.91–3.02)	2.68 (1.73–3.58)	0.82
FEV1 [L]	1.43 (1.16–1.85)	2.03 (1.30–1.57)	0.04	1.50 (1.15–2.12)	1.67 (1.10–2.58)	0.46
FEV1 [%]	49 (44.0–63.0)	64 (50.0–74.0)	0.04	55 (47.0–72.0)	63 (43.0–82.0)	0.72
FEV1/FVC [%]	62.11 (55.22–71.13)	70.43 (64.50–75.63)	0.04	63.28 (56.13–76.06)	70.52 (53.90–4.21)	0.40
ACQ	3.60 (3.20–4.14)	3.66 (3.00–4.40)	0.78	2.54 (1.30–3.30)	2.40 (1.80–3.57)	0.53
miniAQLQ	3.17 (2.10–3.50)	2.60 (2.47–3.17)	0.12	4.08 (3.60–5.80)	3.70 (3.33–4.20)	0.11
OCS [mg]	10 (5.0–20.0)	0 (0.0–10.0)	0.02	0 (0.0–0.0)	0 (0.0–0.0)	0.29
Exacerbation rate	3 (3–5)	3 (2–4)	0.41	0 (0–1)	0 (0–2)	0.82

Point 0—admission for biological therapy; point 1—first follow-up point after 6 months of treatment; CRP—C-reactive protein; ESR—erythrocyte sedimentation rate; FEV1—forced expiratory volume in 1 s; FVC—forced vital capacity; FEV1-FVC—modified Tiffeneau–Pinelli index; ACQ—asthma control questionnaire; miniAQLQ—asthma-related quality of life questionnaire; OCS—oral corticosteroid (dose per prednisone).

## Data Availability

All the obtained and analyzed data are included in this article. Further enquiries can be directed to the corresponding author.
